# A real-world pharmacovigilance analysis of adverse events associated with irbesartan using the FAERS and JADER databases

**DOI:** 10.3389/fphar.2024.1485190

**Published:** 2024-11-20

**Authors:** Qian Liu, Zhiwei Cui, Chao Deng, Chao Yang, Tao Shi

**Affiliations:** ^1^ Department of Cardiovascular Surgery, The First Affiliated Hospital of Xi’an Jiaotong University, Xi’an, Shaanxi, China; ^2^ Department of Obstetrics and Gynecology, The First Affiliated Hospital of Xi’an Jiaotong University, Xi’an, Shaanxi, China

**Keywords:** pharmacovigilance, disproportionality analysis, FAERS, JADER, irbesartan, adverse drug events

## Abstract

**Objective:**

Hypertension is a leading global risk factor for disability and death. Irbesartan, a potent angiotensin II receptor blocker, requires continuous safety monitoring. We conducted a disproportionality analysis of irbesartan-related adverse drug events (ADEs) using the FDA’s FAERS and Japan’s JADER databases.

**Methods:**

We extracted irbesartan-related ADE reports from FAERS (Q1 2004 to Q1 2024) and JADER (Q2 2008 to Q4 2023). We used Reporting Odds Ratio (ROR), Proportional Reporting Ratio (PRR), Bayesian Confidence Propagation Neural Network (BCPNN), and Empirical Bayesian Geometric Mean (EBGM) for signal detection. Sensitivity analyses were conducted to exclude comorbid medications, and subgroup analyses by age and gender were performed to explore ADE occurrence in specific populations. Th time to onset (TTO) of ADEs was assessed using Weibull distribution test and Kaplan-Meier curves.

**Results:**

A total of 5,816 (FAERS) and 366 (JADER) reports were analyzed, with irbesartan-related preferred terms (PTs) involving 27 System Organ Classes (SOCs) in FAERS and 22 in JADER. Three SOCs met detection thresholds in both databases: “metabolism and nutrition disorders,” “cardiac disorders,” and “renal and urinary disorders.” We identified 219 positive signals in FAERS and 20 in JADER, including known signals like hyperkalemia, hypotension, and acute kidney injury. Notably, newly identified signals such as acute pancreatitis (n = 50, ROR: 7.76 [5.88–10.25]) and rhabdomyolysis (n = 50, ROR: 7.76 [5.88–10.25]) in FAERS and respiratory failure (n = 7, ROR: 6.76 [3.20–14.26]) in JADER could have significant clinical implications, as they may lead to severe outcomes if not recognized and managed promptly. Subgroup analyses revealed both similarities and differences in signal detection across gender and age groups. Sensitivity analyses, excluding concomitant medications, confirmed the persistence of key positive signals, including hyperkalemia, angioedema, acute pancreatitis, and agranulocytosis. ADEs mainly occurred within 1 month (34.14%) and after 1 year (32.32%) after dosing, with a median onset of 107 days.

**Conclusion:**

This study provides valuable real-world evidence on the safety profile of irbesartan. The identification of new safety signals underscores the necessity of updating drug labels, particularly for assessing and managing high-risk patients. Additionally, the TTO analysis emphasizes the importance of sustained vigilance for adverse events over time. In conclusion, our findings contribute to a more comprehensive understanding of irbesartan’s safety, aiding healthcare professionals in optimizing its use in clinical practice.

## 1 Introduction

Hypertension, characterized by a sustained systolic blood pressure (SBP) of at least 130 mm Hg or diastolic blood pressure (DBP) of at least 80 mm Hg, significantly elevates the risk of cardiovascular disease events, including coronary heart disease, heart failure, stroke (Cai and Li, 2022), myocardial infarction, and atrial fibrillation, leading to increased mortality and disability worldwide (Carey et al., 2022). Over the past 50 years, advances in pharmacological therapy have significantly improved the safety and efficacy of antihypertensive drugs, particularly those targeting the renin-angiotensin system (RAS), such as angiotensin-converting enzyme inhibitors (ACEIs), angiotensin II receptor blockers (ARBs), and aldosterone antagonists like spironolactone (Cai and Li, 2022; Warwick et al., 2015; Zhou et al., 2021; Robles et al., 2014; Song et al., 2024). Irbesartan, a long-acting angiotensin II type 1 receptor antagonist, is widely approved for hypertension treatment (Morales-Olivas et al., 2004). Irbesartan selectively antagonizes the angiotensin II type 1 (AT1) receptor, effectively inhibiting angiotensin II-induced vasoconstriction and aldosterone secretion. This mechanism promotes vasodilation, reduces sodium and water retention, and consequently lowers blood pressure. Irbesartan is rapidly absorbed orally, with 60%–80% bioavailability unaffected by food, reaching peak blood concentration within 1.5–2 h, and has a plasma protein binding rate of over 90%, primarily metabolized by the liver (Borghi and Cicero, 2012). Compared to enalapril, atenolol, and amlodipine, irbesartan demonstrates superior efficacy in absolute blood pressure reduction and remission rates (Mazzolai et al., 1999). Beyond blood pressure reduction, irbesartan also induces regression of left ventricular hypertrophy, slows the progression of kidney disease in hypertensive patients with type 2 diabetes mellitus, and improves diabetes-related atherosclerosis (Derosa et al., 2009; Chen C. et al., 2018).

Despite irbesartan’s pharmacological benefits, potential safety risks persist in its practical application. Major adverse events reported in epidemiological studies and premarket randomized clinical trials include headache, dizziness, gastric discomfort, skeletal muscle pain, and influenza, with incidence rates varying between 2% and 55% (Morales-Olivas et al., 2004; Kawano et al., 2008; Larochelle et al., 1997; Stumpe et al., 1998; Fogari et al., 1997; Kassler-Taub et al., 1998). In 1,779 patients treated with irbesartan in nine clinical trials, 7.7% reported adverse events within 24 h of the first dose, mainly headache, dizziness and fatigue, with elevated serum creatinine being the most common laboratory abnormality (Simon et al., 1998). A multicenter, randomized, double-blind study of 65-year-old patients with mild-to-moderate hypertension receiving irbesartan therapy showed headache (12.9%), dizziness (4.3%), and arthralgia (2.9%) as the most common non-serious adverse events (Lacourcière, 2000). Although rare, serious adverse effects include hypotension, hyperkalemia, and renal impairment (Hsu et al., 2017). Poor patient compliance due to adverse events remains a major barrier to successful hypertension treatment (Paz Ocaranza et al., 2020). As irbesartan use becomes more widespread, heightened awareness of its safety is essential, particularly for adverse events not explicitly mentioned in drug inserts.

Spontaneous reporting systems are critical for detecting adverse drug events (ADEs) not identified in clinical trials and for conducting safety assessments in specific populations and clinical settings (Noguchi et al., 2021). Recently, the U.S. Food and Drug Administration (FDA) Adverse Event Reporting System (FAERS) and the Japanese Adverse Drug Event Report (JADER) have collected numerous adverse event reports from diverse cohorts (Noguchi et al., 2021; Nomura et al., 2015). These databases are valuable for early detection and identification of potential ADEs, promoting ongoing monitoring and tracking through pharmacovigilance research (Edwards and Biriell, 1994). A study utilizing the WHO pharmacovigilance database (VigiBase), found that patients treated with ARBs reported diarrhea more frequently than those on ACEIs (Guion-Firmin et al., 2022). Additionally, an analysis from the EudraVigilance Data Analysis System (EVDAS) identified a potential association between ARBs, specifically valsartan, and a potential risk of neoplasms (Sharma et al., 2024). Although irbesartan’s safety has been evaluated in clinical trials, stringent inclusion criteria, limited sample sizes, and low incidence of serious ADEs may not capture the full range observed in widespread clinical use (Andersen and Jess, 2014; Luna et al., 2020). Moreover, significant gaps remain in post-marketing pharmacovigilance studies concerning irbesartan’s safety profile in real-world clinical practice. This study aims to address these gaps by providing critical insights into the drug’s safety, focusing on adverse events that may not have been captured in pre-approval trials. This pharmacovigilance study is the first to comprehensively quantify and visualize the safety profile of irbesartan using data from the FAERS and JADER databases, identify new safety signals not previously listed on the drug label, and estimate the timing of ADE occurrence. Our analysis provides valuable information for irbesartan’s clinical practice, guide treatment decisions, and ultimately protect patient health.

## 2 Materials and methods

### 2.1 Data source and collection

FAERS is a publicly accessible database that aggregates and summarizes adverse drug reaction reports from across the globe, providing a comprehensive view of ADE occurrences and facilitating post-marketing safety monitoring. It houses over 9 million individual drug-related adverse event reports submitted by industry professionals, physicians, pharmacists, healthcare professionals, consumers, and others, making it the largest spontaneous reporting system database worldwide (Okada et al., 2019; van Hasselt et al., 2020). The FAERS data is categorized into seven datasets: demographic and administrative information (DEMO), drug information (DRUG), adverse drug reaction information (REAC), patient outcomes information (OUCT), reported sources (RPSR), drug therapy start dates and end dates (THER), and indications for drug administration (INDI). A relationship is established within the FAERS database architecture that connects each data file through a unique identification number (Zhou et al., 2024). Detailed data from FAERS can be downloaded from the FDA website (http://www.fda.gov/).

The JADER database contains information on cases reported by pharmaceutical companies and medical institutions since 2004. The JADER database comprises four files: DEMO, DRUG, REAC, and HIST (Imai et al., 2022). The “DEMO” file includes basic patient information such as sex, age, and weight. The “DRUG” file contains the generic name of the drug, route of administration, and the start and end dates of administration. The “REAC” file records the name of the adverse event, outcome, and the date of occurrence. The “HIST” file contains information about the patient’s underlying condition (Inaba et al., 2019). Data from JADER were downloaded from the Pharmaceutical and Medical Devices Agency website (https://www.pmda.go.jp/index.html).

In our study, we utilized two spontaneous reporting system databases, FAERS and JADER, to mitigate reporting bias and enhance the reliability of the results. Considering the differing marketing times of irbesartan, the analysis period for this study spans from April 2004 to March 2024 in FAERS and from April 2008 to December 2023 in JADER. In FAERS, searches were conducted using generic and brand names such as “IRBESARTAN,” “APROVEL,” and “KARVEA.” In JADER, “イルベサルタン” was used for retrieval. Since FAERS updates quarterly, which may encompass duplicate reports or those that have been withdrawn or deleted, we performed deduplication following FDA recommendations to address duplicate reports submitted by different sources: In the DEMO file, we selected the PRIMARYIDs, CASEIDs, and FDA_DTs, subsequently sorting them by CASEIDs, FDA_DTs, and PRIMARYIDs. (1) If CASEIDs are the same, the latest FDA_DTs is selected; (2) If CASEIDs and FDA_DTs are the same, the higher PRIMARYIDs is selected (Wan et al., 2022). ADE reports associated with irbesartan use were extracted, standardized, and mapped to Medical Dictionary for Regulatory Activities (MedDRA 26.0) (Singh, 2015). The role code for ADEs was retained as the primary suspect (PS) by mitigating drug interactions (I), concomitant drugs (C), secondary suspect drugs, and other unknown drugs that may cause ADEs (Ji et al., 2022; Zhang et al., 2023). Based on MedDRA’s structural hierarchy, the screened ADEs were mapped into preferred terms (PTs) and system organ classes (SOCs). In FAERS, we conducted a statistical analysis of ADE reports, focusing on variables such as sex, age, weight, indication, reporting countries, outcome, and the reported person. During subgroup analyses, we excluded reports with missing data for sex and age. In the JADER database, we removed duplicate data from the DRUG and REAC files and linked the DEMO table to these tables based on the identified conditions (Kato et al., 2022). The drugs were classified into three categories—“PS,” “C,” and “I”—according to their impact on adverse events. Consistent with our approach in FAERS, we designated the role code as PS. Adverse events were coded following the terminology recommended in MedDRA, Japanese version 26.0 (Yamaoka et al., 2022). It is important to note that the DEMO file includes demographic information organized in 10-year age intervals (e.g., 60–69 years). We excluded missing sex data and non-numeric age categories, such as early, mid, late gestation, neonatal, infant, pediatric, young adult, adult, elderly, and unknown, from the subgroup analyses (Kose et al., 2023).

Considering the differences in reporting formats and data structures between FAERS and JADER, we employed the following methods to ensure the comparability of the analysis results:1: Uniform coding system: We utilized a standardized coding system for medical terminology (MedDRA 26.0) to mitigate bias in data analysis stemming from the differing database formats and to ensure the comparability of terms used for adverse reactions across both databases (Zou et al., 2024). 2: Removal of duplicate and erroneous records: We eliminated spontaneous reporting of duplicate or erroneous records within the databases to reduce inconsistencies and to enhance the completeness and credibility of the data analyses. 3: Bayesian algorithms: We employed Bayesian-based algorithms and empirical Bayesian geometric averaging, which effectively address variations in data by adapting to different distributions and structures, thereby generating consistent and reliable signals to ensure comparability of results (Ewers et al., 2012; Agrawal et al., 2019). 4: Timeframe selection: We selected the period following the FDA approval of Irbesartan (September 1997) and its approval in Japan (April 2008) for analysis to minimize bias associated with reporting during the clinical trial phase.

### 2.2 Signal mining and statistical analysis

For pharmacovigilance, disproportionality analysis is commonly employed with post-marketing surveillance databases to assess associations between drugs and adverse events (Shen et al., 2019). This analysis compares the observed and expected number of reports for any given combination of drug and adverse event, generating hypotheses about possible causal relationships. In our study, we applied both Bayesian and frequency (non-Bayesian) methods to evaluate the relationship between irbesartan and ADEs. Frequency methods include Reporting Odds Ratio (ROR) (van Puijenbroek et al., 2002) and Proportional Reporting Ratio (PRR) (Evans et al., 2001). Bayesian methods encompass the Bayesian Confidence Propagation Neural Network (BCPNN) (Bate et al., 1998) and the Multi-Item Gamma Poisson Shrinker (MGPS) (Szarfman et al., 2002). While frequency methods are computationally simple and highly sensitive, they are prone to false positives when the number of adverse events is small (Wu et al., 2023). In contrast, Bayesian methods account for uncertainty in the disproportionality rate when reported cases are limited (Almenoff et al., 2007). In particular, the ROR is more suitable for high-frequency adverse event reporting, offering the advantage of correcting for bias due to a low number of reports for certain events (Fan et al., 2024). In contrast, the PRR is recognized for its higher specificity compared to ROR (Jiang et al., 2024). The BCPNN excels in integrating data from multiple sources and demonstrates robust performance in cross-validation, which helps to reduce the occurrence of false-positive signals. However, it tends to be more conservative, potentially missing signals for very rare events (Lin et al., 2024). In this regard, the MGPS algorithm offers a more comprehensive approach, particularly effective in detecting signals associated with rare events (Li Y. et al., 2024). Each method contributes uniquely to the detection of drug-associated safety signals, and their combined use provides a more balanced and comprehensive understanding of potential drug-adverse event associations (Zou et al., 2023) (Table 1). We define a PT as a positive signal if it simultaneously meets the thresholds of all four methods (Cui et al., 2023). To mitigate the risk of false-positive results (type I errors), we employed the Bonferroni method to adjust for multiple comparisons of *P*-values (Curtin and Schulz, 1998). The adjusted threshold is computed as: Bonferroni-corrected *P*-value = *P*/n, where P is the original significance threshold and n represents the total number of tests conducted. R software (version 4.2.1) was used for data processing and statistical analysis.

**TABLE 1:** Methods and thresholds for ROR, PRR, BCPNN, and EBGM (Wang et al., 2024b; Li R. et al., 2024).

	Target adverse drug event	Other adverse drug events	Sums
Irbesartan	a	b	a+b
Other drugs	c	d	c + d
Sums	a+c	b + d	a+b + c + d

Algorithms	Equation	Criteria
ROR PRR BCPNN MGPS	ROR = ad/bc 95%CI = e^ln(ROR)±1.96(1/a+1/b+1/c+1/d)^0.5^ PRR = [a (c + d)]/[c (a+b)] χ^2^ = [(ad-bc)^2](a+b + c + d)/[(a+b) (c + d) (a+c) (b + d)] IC = log_2_a (a+b + c + d)/[(a+c) (a+b)] 95%CI = E (IC) ± 2 [V(IC)]^0.5 EBGM = a (a+b + c + d)/[(a+c) (a+b)] 95%CI = e^ln(EBGM)±1.96(1/a+1/b+1/c+1/d)^0.5^	lower limit of 95% CI > 1, a≥3 PRR≥2, χ^2^ ≥ 4, a≥3 IC025 > 0 EBGM05 > 2

a: count of reports with both specified drug and target adverse events; b: reports involving other adverse drug events with the specified drug; c: reports of target adverse drug events involving other drugs; d: reports encompassing other drugs and non-targeted adverse drug events. ROR: reporting odds ratio, PRR: proportional reporting ratio, BCPNN: Bayesian confidence propagation neural network, EBGM: empirical bayesian geometric mean, 95% CI: 95% confidence interval; N: number of reports; χ2: chi-squared; IC: information component; IC025: lower limit of 95% CI of the IC; E (IC): IC expectations; V(IC): variance of IC; EBGM05: lower limit of 95% CI of EBGM.

### 2.3 Time to onset analysis

The time to onset (TTO) of irbesartan-related ADEs was defined as the interval between EVENT_DT (date of ADE onset in the DEMO file) and START_DT (date of medication initiation in the THER file). Cases with missing dates (either the initiation of medication or the onset of ADEs) or inaccuracies (not specified to a particular day, month, or year) were excluded. Additionally, cases in which the onset date of ADE occurred before the initiation date of irbesartan therapy were also excluded, as this would result in a negative time-to-onset calculation (Zou et al., 2024). We employed the median, quartile, minimum, maximum, and Weibull shape parameter to comprehensively assess TTO characteristics in our study (Kinoshita et al., 2020). The Kaplan-Meier method was employed to illustrate the cumulative incidence of ADEs associated with irbesartan (Wang et al., 2024). The frequency of adverse events following the initiation of treatment is contingent upon the drug’s mechanism of action and may vary over time. In contrast, the incidence of adverse events not associated with drug therapy tends to be more stable (Cornelius et al., 2012). Changes in the risk incidence of ADEs over time can be identified and predicted by the Weibull distribution test, with scale (α) and shape (β) parameters characterizing the Weibull distribution’s shape (Mazhar et al., 2021). In this study, we focus exclusively on the parameter β. When the shape parameter β is less than 1 and its 95% confidence interval (CI) is also below 1, the risk of adverse effects is considered to decrease over time, indicative of an early failure-type curve. Conversely, if β is approximately equal to 1, with its 95% CI containing the value of 1, the risk is estimated to remain persistent over time, representing a random failure-type curve. Finally, if β is greater than 1 and its 95% CI does not encompass the value of 1, the hazard is interpreted as increasing over time, characteristic of a wear-out failure-type curve (Mazhar et al., 2021; Sauzet et al., 2013).

## 3 Results

### 3.1 Basic characteristics of ADE reports

**FIGURE 1:**
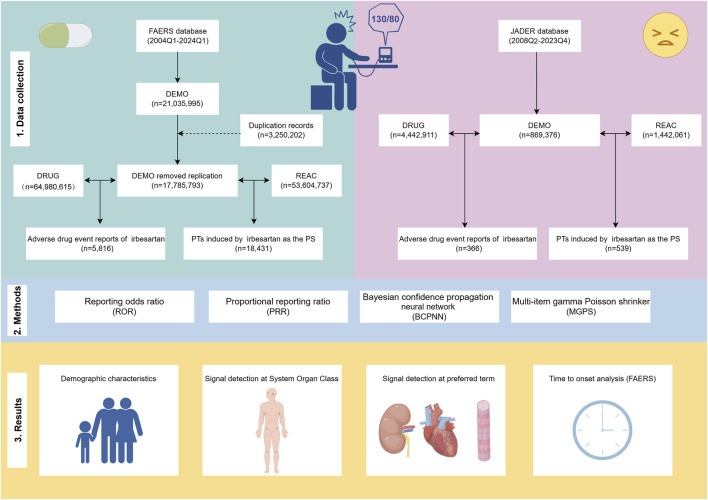
Flowchart of the research. The study comprises data collection and cleaning (Section 1), disproportionality analysis methods to calculate signal strength (Section 2), and presentation of results (Section 3). FAERS: FDA Adverse Event Reporting System, JADER: Japanese Adverse Drug Event Report, Q1: first quarter, Q4: fourth quarter, PT: preferred term, PS: primary suspect.

**FIGURE 2:**
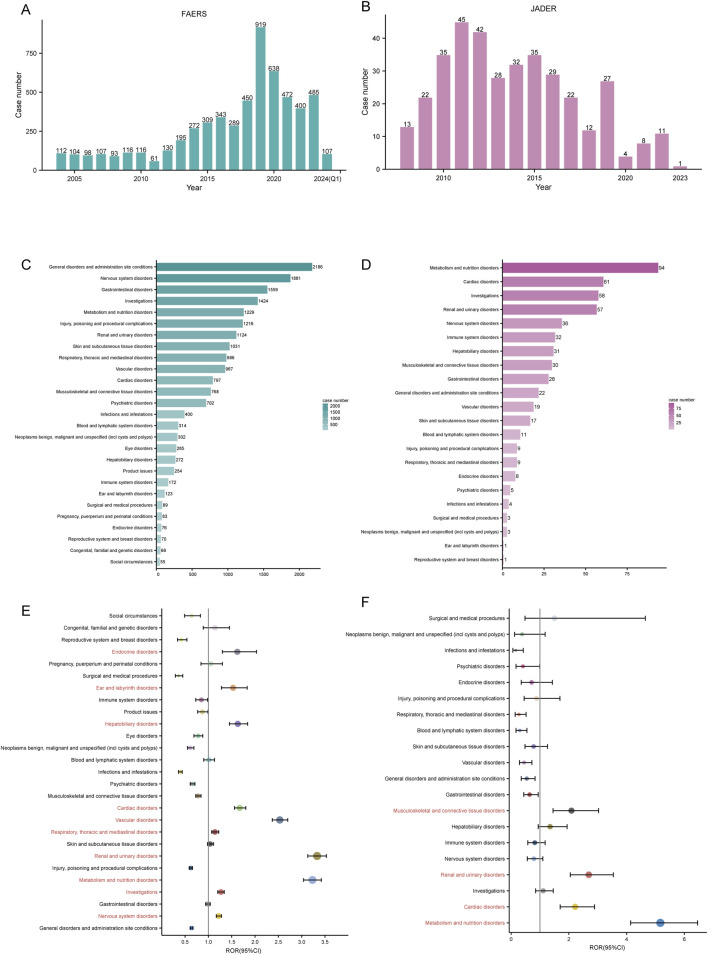
Signal detection at the SOC level. Annual ADE reports in the FAERS **(A)** and JADER **(B)** databases are shown as bar charts. The number of irbesartan-induced ADEs at the SOC level in FAERS **(C)** and JADER **(D)**. Signal detection at the SOC level in FAERS **(E)** and JADER **(F)**. ROR values and their 95% confidence intervals (95% CI) are visualized. SOC: System Organ Class, ADE: adverse drug event, FAERS: FDA Adverse Event Reporting System, JADER: Japanese Adverse Drug Event Report, ROR: reporting odds ratio.

A total of 5,816 and 366 ADE reports were obtained from the FAERS (January 2004-January 2024) and JADER (April 2008-December 2023) databases, respectively (Figure 1). The number of ADE reports in the FAERS database remained above 400 annually from 2018 through 2023 (Figure 2A), whereas in JADER, the number of reports did not exceed 50 per year (Figure 2B).

The characteristics of ADEs reported in both databases, including age, weight, sex, outcome, indication, and reported country, are detailed in Tables 2, 3. In FAERS, ADEs were more frequently reported in female patients (n = 2,916, 50.1%) than male patients (n = 2,095, 36.0%). Among the known age reports, patients aged 65 years or older constituted a major portion (n = 2,878, 49.5%). Body weight, a critical indicator for evaluating adverse reactions to chemical drugs (Yuan et al., 2020), was missing for most patients (n = 4,075, 70.1%). Most reports were submitted by health professionals (n = 3,707, 63.8%), with serious adverse outcomes mainly being other-serious medical events (n = 2,797, 38.2%) and hospitalization-initial or prolonged (n = 2,682, 36.6%). Notably, the proportion of deaths and life-threatening events reported was 3.6% and 5.5%, respectively. The primary indication for irbesartan use was hypertension (n = 2,997, 51.5%), with other indications including essential hypertension (n = 74, 1.2%) and cardiac failure (n = 57, 0.9%) (Table 2).

**TABLE 2:** Demographic characteristics of ADEs reported in the FAERS database with irbesartan as the primary suspect drug.

Characteristics	Case number	Case proportion, %
Sex, n (%)		
Female	2916	50.1%
Male	2095	36.0%
Unknown	805	13.9%
Age		
<18 years	28	0.5%
18–64 years	1286	22.1%
≥65 years	2878	49.5%
Unknown	1624	27.9%
Weight		
<50 kg	110	1.9%
50–100 kg	1450	3.1%
>100 kg	181	24.9%
Unknown	4075	70.1%
Reported Countries (top five)		
France	1978	24.2%
United States	1044	12.8%
United Kingdom	602	10.2%
Italy	344	8.5%
Canada	334	6.0%
Reported person		
Health professional	3707	63.8%
Consumer	2021	34.7%
Unknown	88	1.5%
Outcome		
Hospitalization-initial or prolonged	2682	36.6%
Life-threatening	401	5.5%
Disability	203	2.8%
Required intervention	12	0.2%
Death	260	3.6%
Other serious outcomes	2797	38.2%
Congenital anomaly	29	0.4%
Unknown	938	12.8%
Indication (top five)		
Hypertension	2997	51.5%
Blood pressure measurement	84	1.4%
Essential hypertension	74	1.2%
Cardiac failure	57	0.9%
Blood pressure	55	0.9%

FAERS: FDA adverse event reporting system.

**TABLE 3:** Demographic characteristics of ADEs reported in the JADER database with irbesartan as the primary suspect drug.

Characteristics	Case number	Case proportion, %
Sex, n (%)		
Female	152	41.5%
Male	204	55.7%
Unknown	10	2.7%
Age		
<65 years	135	36.9%
≥65 years	212	57.9%
Unknown	19	5.2%
Weight		
<50 kg	50	13.7%
50–100 kg	158	43.2%
>100 kg	2	0.5%
Unknown	156	42.6%
Outcome		
Recovery (recovery but with sequelae)	13	2.4%
Rehabilitation	211	39.1%
Minor rehabilitation	148	27.5%
Death	28	5.2%
Non-rehabilitated	56	10.4%
Missing	83	15.4%
Indication (top three)		
Hypertension	291	72.6%
Gestational hypertension	3	0.7%
Lupus nephritis	2	0.5%

JADER: japanese adverse drug event report.

Conversely, in JADER, higher proportions of male patients (n = 204, 55.7%) submitted more ADE reports compared to female patients (n = 152, 41.5%). The number of reported cases for individuals under 65 years of age is 135, accounting for 36.9% of the total reports. In contrast, the number of reports for individuals aged 65 years or older is 212, representing 57.9% of the total. Among those who provided specific body weight information, the largest number of reports were in the 50–100 kg weight range (n = 158, 43.2%). Sixty nine percent of the submitters experienced varying degrees of recovery or rehabilitation, but 5.2% of the submitters died. Consistent with FAERS reports, the primary indication for irbesartan was hypertension (n = 291, 72.6%) (Table 3).

### 3.2 Signal detection at the SOC level

Mapping the PTs in ADE reports to the corresponding SOC level, we counted the number of reports in both databases. We ranked the case number of PTs in descending order for each SOC and found that irbesartan-associated PTs mainly involved 27 SOCs in FAERS and 22 SOCs in JADER. The top 3 SOCs in terms of reported cases differed between the databases. In FAERS, they were general disorders and administration site conditions (n = 2,186), nervous system disorders (n = 1,881), and gastrointestinal disorders (n = 1,559) (Figure 2C). In JADER, they were metabolism and nutrition disorders (n = 94), cardiac disorders (n = 61), and investigations (n = 58) (Figure 2D). However, among the top 5 SOCs, three overlapped: nervous system disorders, metabolism and nutrition disorders, and investigations.

The signal strength of ADE reports in both databases at the SOC level was calculated using disproportionality analysis. Based on the ROR method, we plotted the forest of signal strength. In FAERS, ten SOCs met the ROR positive threshold: nervous system disorders (SOC code: 10029205), investigations (SOC code: 10022891), metabolism and nutrition disorders (SOC code: 10027433), renal and urinary disorders (SOC code: 10038359), respiratory, thoracic and mediastinal disorders (SOC code: 10038738), vascular disorders (SOC code: 10047065), cardiac disorders (SOC code: 10007541), hepatobiliary disorders (SOC code: 10019805), ear and labyrinth disorders (SOC code: 10013993), and endocrine disorders (SOC code: 10014698) (Figure 2E). In JADER, four SOCs met the positive threshold: metabolism and nutrition disorders, cardiac disorders, renal and urinary disorders, and musculoskeletal and connective tissue disorders (SOC code: 10028395) (Figure 2F). The three SOCs positive in both databases were metabolism and nutrition disorders, cardiac disorders, and renal and urinary disorders.

Notably, in FAERS, the signal strength of renal and urinary disorders (ROR: 3.33 [3.13–3.53], PRR: 3.19, EBGM05: 3.00, IC025: 0.01) met the positive threshold for all four disproportionality methods (Table 4). In JADER, metabolism and nutrition disorders (ROR: 5.17 [4.14–6.46], PRR: 4.45, EBGM05: 3.55, IC025: 0.48) was the only SOC that simultaneously met the positivity threshold for all four methods (Table 5). The complete results are presented in Tables 4, 5.

**TABLE 4:** Signal detection at the SOC level in FAERS.

System Organ Class	SOC code	Case number	ROR(95%CI)	PRR(χ2)	EBGM(EBGM05)	IC(IC025)
General disorders and administration site conditions	10017581	2186	0.64(0.61–0.67)	0.68(396.08)	0.68(0.65)	−0.55(−2.22)
Nervous system disorders	10000346	1881	1.22(1.17–1.28)	1.2(68.48)	1.2(1.14)	0.26(−1.4)
Gastrointestinal disorders	10000050	1559	0.99(0.94–1.04)	0.99(0.1)	0.99(0.94)	−0.01(−1.68)
Investigations	10063264	1424	1.27(1.2–1.34)	1.25(73.82)	1.25(1.18)	0.32(−1.35)
Metabolism and nutrition disorders	10065941	1229	3.23(3.04–3.42)	3.08(1760.26)	3.08(2.9)	1.62(−0.05)
Injury, poisoning and procedural complications	10000044	1216	0.62(0.59–0.66)	0.65(263.25)	0.65(0.61)	−0.63(−2.3)
Renal and urinary disorders	10073515	1124	3.33(3.13–3.53)	3.19(1716.22)	3.18(3)	1.67(0.01)
Skin and subcutaneous tissue disorders	10058820	1031	1.04(0.98–1.11)	1.04(1.87)	1.04(0.98)	0.06(−1.61)
Respiratory, thoracic and mediastinal disorders	10051545	986	1.14(1.07–1.22)	1.14(16.75)	1.14(1.06)	0.18(−1.48)
Vascular disorders	10000358	967	2.53(2.37–2.7)	2.45(848.18)	2.45(2.3)	1.29(−0.37)
Cardiac disorders	10077162	797	1.67(1.56–1.8)	1.64(206.26)	1.64(1.53)	0.72(−0.95)
Musculoskeletal and connective tissue disorders	10074599	768	0.78(0.73–0.84)	0.79(43.41)	0.79(0.74)	−0.33(−2)
Psychiatric disorders	10082331	702	0.66(0.61–0.71)	0.67(119.23)	0.67(0.62)	−0.57(−2.24)
Infections and infestations	10060921	400	0.4(0.36–0.44)	0.41(354.73)	0.41(0.37)	−1.28(−2.95)
Blood and lymphatic system disorders	10073485	314	1.01(0.9–1.13)	1.01(0.04)	1.01(0.9)	0.02(−1.65)
Neoplasms benign, malignant and unspecified (incl cysts and polyps)	10068532	302	0.61(0.55–0.69)	0.62(73.31)	0.62(0.55)	−0.69(−2.36)
Eye disorders	10000173	285	0.78(0.69–0.88)	0.78(17.23)	0.78(0.7)	−0.35(−2.02)
Hepatobiliary disorders	10071634	272	1.63(1.45–1.84)	1.62(65.18)	1.62(1.44)	0.7(−0.97)
Product issues	10078577	254	0.87(0.77–0.99)	0.87(4.73)	0.87(0.77)	−0.2(−1.86)
Immune system disorders	10000206	172	0.85(0.73–0.98)	0.85(4.79)	0.85(0.73)	−0.24(−1.91)
Ear and labyrinth disorders	10063559	123	1.53(1.28–1.83)	1.53(22.65)	1.53(1.28)	0.61(−1.05)
Surgical and medical procedures	10059486	89	0.36(0.29–0.45)	0.37(99.45)	0.37(0.3)	−1.45(−3.12)
Pregnancy, puerperium and perinatal conditions	10084854	83	1.05(0.84–1.3)	1.05(0.16)	1.05(0.84)	0.06(−1.6)
Endocrine disorders	10080230	76	1.62(1.3–2.03)	1.62(18.11)	1.62(1.29)	0.7(−0.97)
Reproductive system and breast disorders	10087591	70	0.42(0.34–0.54)	0.43(54.58)	0.43(0.34)	−1.23(−2.9)
Congenital, familial and genetic disorders	10000002	66	1.14(0.89–1.45)	1.14(1.11)	1.14(0.89)	0.19(−1.48)
Social circumstances	10000209	55	0.64(0.49–0.83)	0.64(11.07)	0.64(0.49)	−0.64(−2.31)

FAERS: FDA Adverse Event Reporting System, ROR: reporting odds ratio; CI: confidence interval; PRR: proportional reporting ratio; χ2: chi-squared; IC: information component; IC025: lower limit of the 95% CI of IC; EBGM: empirical Bayes geometric mean, EBGM05: lower limit of the 95% CI of EBGM, SOC: System Organ Class.

**TABLE 5:** Signal detection at the SOC level in JADER.

System Organ Class	SOC code	Case number	ROR (95%CI)	PRR (χ2)	EBGM (EBGM05)	IC (IC025)
Metabolism and nutrition disorders	10065941	94	5.17(4.14–6.46)	4.45(260.87)	4.44(3.55)	2.15(0.48)
Cardiac disorders	10077162	61	2.22(1.7–2.89)	2.08(36.05)	2.08(1.59)	1.05(−0.62)
Investigations	10063264	58	1.11(0.85–1.46)	1.1(0.6)	1.1(0.84)	0.14(−1.53)
Renal and urinary disorders	10073515	57	2.69(2.05–3.54)	2.51(54.14)	2.51(1.91)	1.33(−0.34)
Nervous system disorders	10000346	36	0.79(0.56–1.1)	0.8(1.95)	0.8(0.57)	−0.32(−1.99)
Immune system disorders	10000206	32	0.82(0.58–1.18)	0.83(1.13)	0.83(0.58)	−0.26(−1.93)
Hepatobiliary disorders	10071634	31	1.35(0.94–1.94)	1.33(2.67)	1.33(0.93)	0.41(−1.26)
Musculoskeletal and connective tissue disorders	10074599	30	2.09(1.45–3.03)	2.03(16.18)	2.03(1.41)	1.02(−0.65)
Gastrointestinal disorders	10000050	28	0.64(0.44–0.94)	0.66(5.36)	0.66(0.45)	−0.6(−2.27)
General disorders and administration site conditions	10017581	22	0.54(0.35–0.83)	0.56(8.28)	0.56(0.36)	−0.84(−2.51)
Vascular disorders	10000358	19	0.45(0.29–0.72)	0.47(11.99)	0.47(0.3)	−1.08(−2.75)
Skin and subcutaneous tissue disorders	10058820	17	0.78(0.48–1.26)	0.79(1.03)	0.79(0.49)	−0.35(−2.02)
Blood and lymphatic system disorders	10073485	11	0.3(0.17–0.55)	0.32(17.5)	0.32(0.17)	−1.67(−3.34)
Respiratory, thoracic and mediastinal disorders	10051545	9	0.27(0.14–0.52)	0.28(17.37)	0.28(0.15)	−1.82(−3.49)
Injury, poisoning and procedural complications	10000044	9	0.88(0.45–1.69)	0.88(0.16)	0.88(0.45)	−0.19(−1.86)
Endocrine disorders	10080230	8	0.71(0.35–1.43)	0.72(0.91)	0.72(0.36)	−0.48(−2.15)
Psychiatric disorders	10082331	5	0.41(0.17–0.99)	0.42(4.21)	0.42(0.17)	−1.27(−2.94)
Infections and infestations	10060921	4	0.16(0.06–0.42)	0.16(17.87)	0.16(0.06)	−2.61(−4.28)
Neoplasms benign, malignant and unspecified (incl cysts and polyps)	10068532	3	0.38(0.12–1.18)	0.38(3.05)	0.38(0.12)	−1.39(−3.06)
Surgical and medical procedures	10059486	3	1.5(0.48–4.65)	1.49(0.49)	1.49(0.48)	0.58(−1.09)

JADER: Japanese Adverse Drug Event Report, ROR: reporting odds ratio; CI: confidence interval; PRR: proportional reporting ratio; χ2: chi-squared; IC: information component; IC025: lower limit of the 95% CI of IC; EBGM: empirical Bayes geometric mean, EBGM05: lower limit of the 95% CI of EBGM, SOC: System Organ Class.

### 3.3 Signal detection at the PT level

**FIGURE 3:**
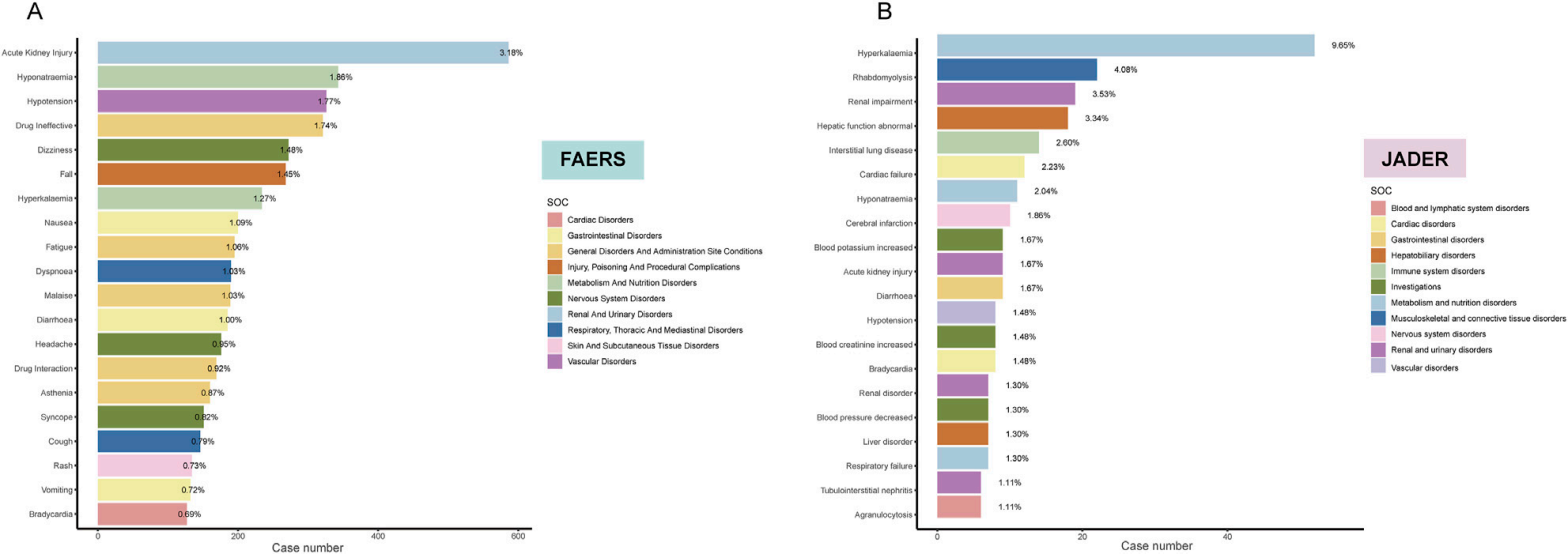
Bar plot illustrating the top 20 PT statistics in FAERS **(A)** and JADER **(B)** databases. The color indicates the SOC of the corresponding PT. Percentage values represent the proportion of cases with such ADEs out of the total reported ADEs.

Subsequently, considering only the frequency of reports, we identified the top 20 PTs in both cohorts. In FAERS, the PT with the highest number of reported cases was acute kidney injury (n = 586, 3.18%), followed by hyponatremia (n = 343, 1.86%), hypotension (n = 326, 1.77%), drug ineffective (n = 321, 1.74%), and dizziness (n = 272, 1.48%) (Figure 3A). In contrast, the top 5 PTs in the JADER database were hyperkalemia (n = 52, 9.65%), rhabdomyolysis (n = 22, 4.08%), renal impairment (n = 19, 3.53%), hepatic function abnormal (n = 18, 3.34%), and interstitial lung disease (n = 14, 2.60%) (Figure 3B). Additionally, we identified six overlapping signals: acute kidney injury, hyponatremia, hypotension, hyperkalemia, diarrhea, and bradycardia.

**FIGURE 4:**
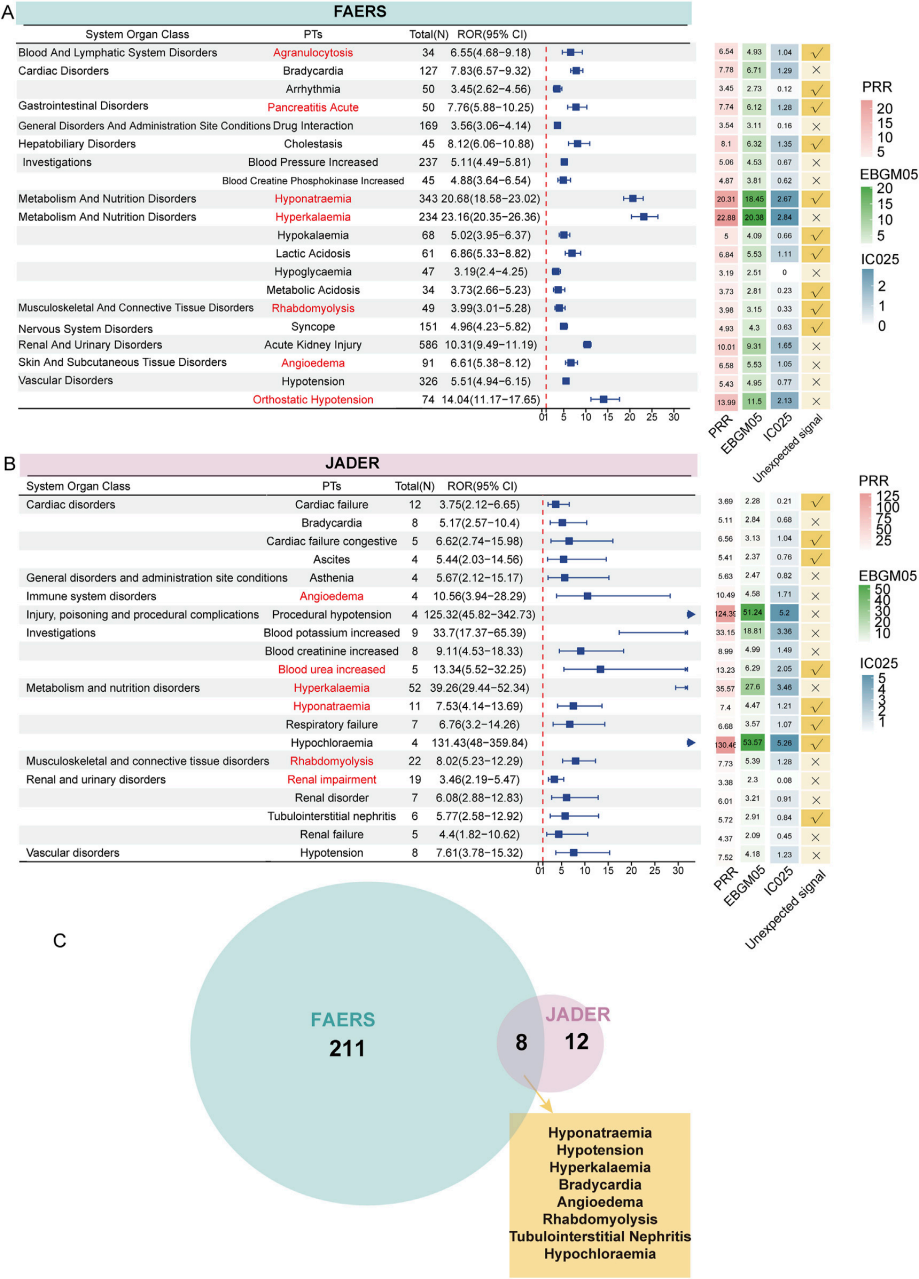
Signal detection at the PT level. The forest plot presents the ROR values along with their confidence intervals for the top 20 positive signals (ranked by case number) associated with irbesartan at the PT level in FAERS **(A)** and JADER **(B)** databases. These PTs are categorized by SOC. Adjacent to this, the heatmap illustrates the PRR, EBGM05, and IC025 values for these signals, offering a comprehensive visual of signal strength across different metrics. Arrows in the forest plot indicate instances where the lower limit of the ROR exceeds 30, signifying a notably strong association between irbesartan and the specific adverse event signals at the PT level. Signals of interest have been highlighted in red for emphasis. Venn diagram showing the overlap of 219 positive signals in FAERS and 20 positive signals in JADER **(C)**. ROR: Reporting Odds Ratio; PRR: Proportional Reporting Ratio, EBGM05: the lower limit of the 95% CI of EBGM, IC025: the lower limit of the 95% CI of the IC, SOC: System Organ Class, PT: preferred term, FAERS: FDA Adverse Event Reporting System, JADER: Japanese Adverse Drug Event Report.

Using disproportionality methods, we calculated the signal strength of each PT and filtered out all signals that simultaneously met the positivity thresholds. In FAERS, 219 signals met the criteria, while in JADER, 20 signals were identified. Based on the descending order of filtered signals from the reported cases and grouped according to SOC, we presented the ROR values and their 95% CIs for the top 20 signals in each cohort using a forest plot. Generally, higher ROR values indicate a stronger association of these signals with irbesartan. In FAERS, several PTs had a high number of reports along with relatively strong signal strength, including hyponatremia (n = 343, ROR: 20.68 [18.58–23.02], PRR: 20.31, EBGM05: 18.45, IC025: 2.67), hyperkalemia (n = 234, ROR: 23.16 [20.35–26.36], PRR: 22.88, EBGM05: 20.38, IC025: 2.84), and orthostatic hypotension (n = 74, ROR: 14.04 [11.17–17.65], PRR: 13.99, EBGM05: 11.50, IC025: 2.13). Despite the small number of cases, some signals demonstrated strong signal strength, including amyloid arthropathy (n = 11, ROR: 4361.81 [728.77–1602.36], PRR: 820.04, EBGM05: 365.48, IC025: 7.61), neurologic neglect syndrome (n = 22, ROR: 156.19 [101.69–239.91], PRR: 156.01, EBGM05: 148.11, IC025: 5.54), carotid artery thrombosis (n = 24, ROR: 108.66 [72.27–163.36], PRR: 108.52, EBGM05: 74.40, IC025: 5.04), and personality disorder (n = 24, ROR: 30.10 [20.13–45.01], PRR: 30.06, EBGM05: 21.26, IC025: 3.23) (Supplementary Table S1). Notably, we identified several new signals not listed in the drug label, including hyponatremia, syncope, lactic acidosis, arrhythmia, acute pancreatitis, rhabdomyolysis, and cholestasis (Figure 4A). Supplementary Table S1 provides the full results of the analysis in FAERS.

In the JADER database, the filtered 20 signals are listed in Figure 4B. PTs with a high number of reported cases included hyperkalemia (n = 52, ROR: 39.26 [29.44–52.34], PRR: 35.57, EBGM05: 27.60, IC025: 3.46), rhabdomyolysis (n = 22, ROR: 8.02 [5.23–12.29], PRR: 7.73, EBGM05: 5.39, IC025: 1.28), and renal impairment (n = 19, ROR: 3.46 [2.19–5.47], PRR: 3.38, EBGM05: 2.30, IC025: 0.08). PTs with strong signal strengths included hyperkalemia, increased blood potassium (n = 9, ROR: 33.70 [17.37–65.39], PRR: 33.15, EBGM05: 18.81, IC025: 3.36), procedural hypotension (n = 4, ROR: 125.32 [45.82–342.73], PRR: 124.39, EBGM05: 51.24, IC025: 5.20), and hypochloremia (n = 4, ROR: 131.43 [48.00–359.84], PRR: 130.46, EBGM05: 53.57, IC025: 5.26). New signals included cardiac failure (n = 12, ROR: 3.75 [2.12–6.65], PRR: 3.69, EBGM05: 2.28, IC025: 0.21), hyponatremia (n = 11, ROR: 7.53 [4.14–13.69], PRR: 7.40, EBGM05: 4.47, IC025: 1.21), respiratory failure (n = 7, ROR: 6.76 [3.20–14.26], PRR: 6.68, EBGM05: 3.57, IC025: 1.07), tubulointerstitial nephritis (n = 6, ROR: 5.77 [2.58–12.92], PRR: 5.72, EBGM05: 2.91, IC025: 0.84), and blood urea increased (n = 5, ROR: 13.34 [5.52–32.25], PRR: 13.23, EBGM05: 6.29, IC025: 2.05) (Figure 4B). Supplementary Table S2 provides the full results of the analysis in JADER.

Combining the results from both databases, we identified eight positive new signals screened in both databases: hyponatremia, hypotension, hyperkalemia, bradycardia, angioedema, rhabdomyolysis, tubulointerstitial nephritis, and hypochloremia (Figure 4C). To visually represent the most significant ADE signals, volcano plots were generated for the analysis results of both FAERS (Supplementary Figure S1A) and JADER (Supplementary Figure S1B) databases. The plots display 219 and 20 positive signals, respectively. In these plots, the horizontal axis represents the log2-transformed ROR values, while the vertical axis shows the -log10-transformed corrected *P*-values (adjusted using the Bonferroni method). Signals on the right side of the plot, corresponding to higher log2-transformed ROR values, indicate a stronger association with irbesartan compared to those on the left. Strong positive signals identified in FAERS include rare events such as atrial standstill and amyloid arthropathy. In the JADER database, notable signals include hypochloremia and procedural hypotension. These signals represent adverse drug events that show a strong association with irbesartan based on the data from these pharmacovigilance databases, further highlighting the importance of monitoring specific reactions in different populations.

### 3.4 Subgroup analysis

To minimize the influence of confounding factors, we conducted subgroup analyses of adverse events associated with irbesartan using data from both the FAERS and JADER databases. The top 15 most common adverse events for each subgroup were identified based on positive signal criteria (Supplementary Figures S2A, C, E, G). In FAERS, rhabdomyolysis and acute pancreatitis were male-specific, while aphasia and arrhythmia were female-specific (Supplementary Figure S2B). Signals such as muscle spasticity, urinary retention, and dysphagia were predominant in patients under 65, while bradycardia, eczema, and orthostatic hypotension were observed in those 65 and older (Supplementary Figure S2D). In JADER, four overlapping signals—hyperkalemia, rhabdomyolysis, blood potassium increased, and blood creatinine increased were found in both male and female subgroups (Supplementary Figure S2F). Age-specific signals included renal impairment and hepatic function abnormal in the age less than 65 subgroup, and agranulocytosis and respiratory failure in those aged 65 and older (Supplementary Figure S2H).

### 3.5 Sensitivity analysis

In clinical practice, irbesartan is often co-administered with other antihypertensive agents such as hydrochlorothiazide and amlodipine to enhance blood pressure control. To eliminate the potential effects of concomitant medications on our results, we conducted a sensitivity analysis. After excluding cases with co-administration of other drugs, we identified 1,479 reports. Persistent adverse reactions included hyponatraemia, hyperkalemia, angioedema, acute pancreatitis, arrhythmia, increased blood creatine phosphokinase, presyncope, swollen tongue, and agranulocytosis, among others (Supplementary Table S3).

### 3.6 Time to onset analysis

**FIGURE 5:**
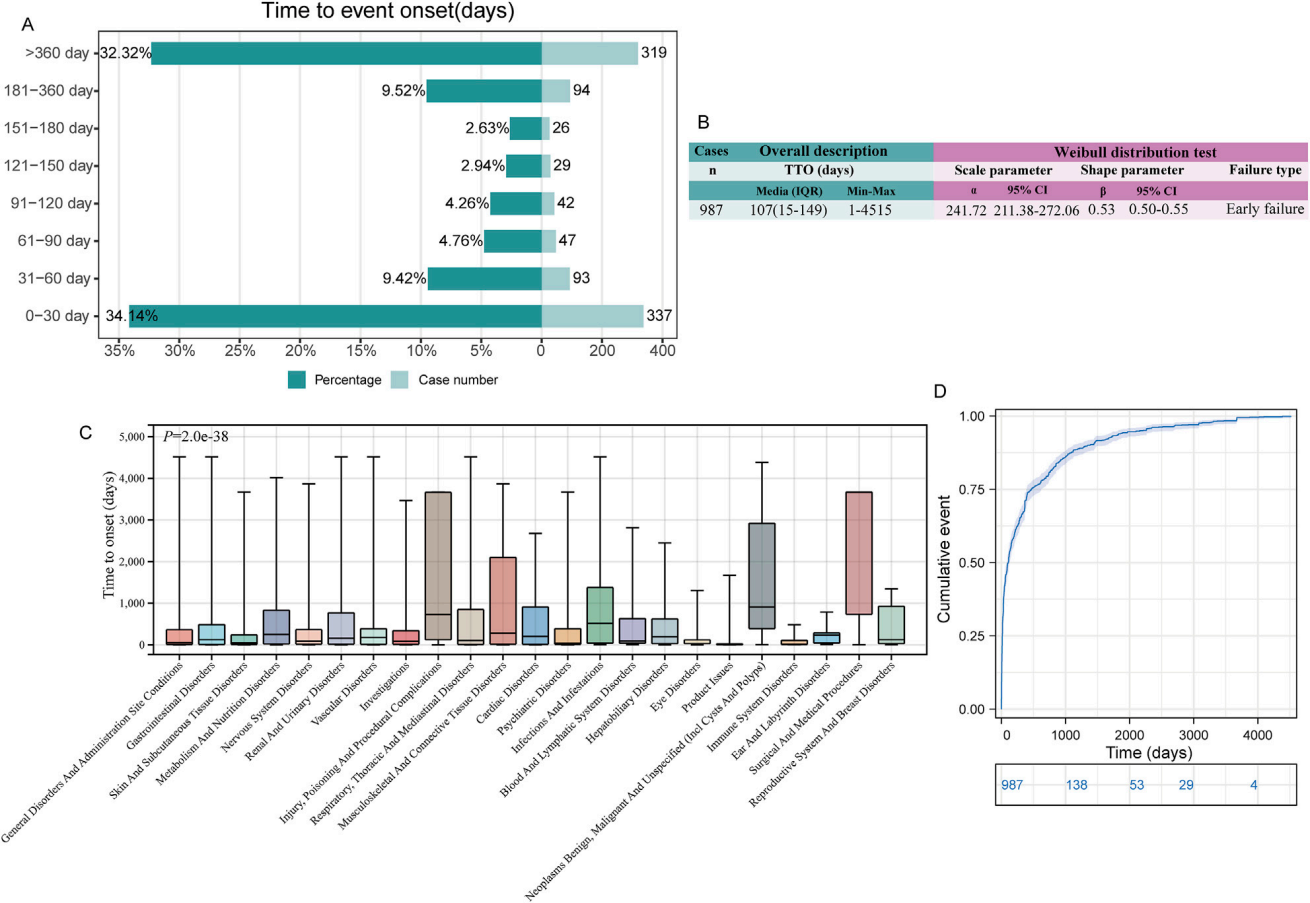
Time to onset (TTO) analysis (in days) of irbesartan-related ADEs. **(A)**. Bar graphs depict the number and proportion of ADE reports at different time intervals. **(B)**. Overall description and Weibull distribution test analysis of effective TTO reports. The overall analysis highlights the median occurrence of ADEs along with the maximum and minimum values across all TTO reports. The results from the Weibull distribution analysis are presented in terms of scale and shape parameters, which describe the time-dependent risk patterns of ADEs. The scale parameter provides insight into the timeframe of event occurrence, while the shape parameter indicates whether the risk of ADE increases, decreases, or remains constant over time. **(C)**. Box plot of the TTO at the SOC level. The bold bar within the box represents the median TTO, while the lower and upper ends of the box denote the 1st and 3rd quartiles, respectively, indicating the interquartile range. **(D)**. The Kaplan-Meier curve depicts the cumulative incidence of TTO occurrence over time, providing a visual representation of the probability of event occurrence across different time intervals. ADE: adverse drug event; IQR: interquartile range.

Due to the limited number of effective TTO reports in the JADER database, we only statistically analyzed the TTO reports in the FAERS database. In FAERS, there were 987 (17.0%) total effective TTO reports. The median onset time of all ADEs was 107 days, with an interquartile range (IQR) of 15–469 days (Figure 5B).

ADEs following irbesartan administration occurred primarily within 1 month of administration (n = 337, 34.14%) and after 1 year of administration (n = 319, 32.32%). Within 180 days, the number of TTO reports tended to decrease over time, but reports beyond 180 days still accounted for about 40% of cases (Figure 5A). The Weibull distribution test for TTO indicated that the upper limit of the 95% confidence interval (CI) for the shape parameter (β) was less than 1 in FAERS (0.55), indicating an early failure type, suggesting that the probability of an ADE gradually decreased over time (Figure 5B).

Additionally, we analyzed the TTO reports at the SOC level. Among the 23 SOC levels with at least 10 valid TTO reports, there was a significant difference in TTOs (*P* < 0.0001, Figure 5C). SOCs with the shortest median onset times included “product issues” [median onset time (MOT): 10 days], “immune system disorders” (MOT: 17 days), and “eye disorders” (MOT: 31 days). SOCs with the longest median TTOs included “surgical and medical procedures” (MOT: 3,667 days), “neoplasms benign, malignant, and unspecified (incl. cysts and polyps)” (MOT: 911 days), and “injury, poisoning, and procedural complications” (MOT: 730 days) (Supplementary Table S4). The cumulative incidence of ADEs over time is depicted in a Kaplan-Meier plot (Figure 5D).

## 4 Discussion

### 4.1 Baseline information description

Our analysis of baseline information revealed notable differences in the demographic data concerning irbesartan ADEs between the FAERS and JADER databases. In FAERS, the majority of ADE reports were submitted by females (50.1%) compared to males (36.0%), with 49.5% of submitters aged over 65 years. Despite a significant portion of reports lacking specific weight information, weights over 100 kg represented a major segment of the known weights. The primary reporting regions were the Americas (United States, Canada) and Europe (France, United Kingdom, and Italy), constituting 61.7% of the reports.

Conversely, in JADER, the proportion of male submitters (55.7%) exceeded that of females (41.5%). While 57.9% of the submitters were aged 65 years or older, 36.9% were under 65 years of age, and weights fell primarily within the 50–100 kg range (43.2%), with the vast majority of reports originating from Japan. These demographic variations can be partially attributed to the epidemiological characteristics of hypertension. In 2016, 34.6% of Japanese men and 24.8% of Japanese women were reported to suffer from hypertension (Asakura et al., 2021). By 2017, the prevalence of hypertension among the Japanese working-age population (20–64 years) was 37.5%. Notably, mean systolic and DBP levels have decreased significantly in middle-aged and older Japanese adults due to advancements in hypertension treatment (Hisamatsu et al., 2020). Examining trends over a 55-year period from 1961 to 2016, DBP levels in women decreased by 4–8 mm Hg across all age groups. However, in men aged 50–59, DBP levels remained unchanged, and in men aged 30–49, they increased, potentially due to rising obesity rates, reduced physical activity, and inadequate diastolic hypertension treatment (Nagai et al., 2015; Hisamatsu and Miura, 2024). Similarly, a report from the American Heart Association indicates that in the United States, the prevalence of hypertension is increasing twice as fast in women compared to men, with older women (≥65 years) showing higher prevalence rates than men (Mozaffarian et al., 2015). This trend may be related to post-menopausal hormonal changes, activation of the RAS system, sympathetic nervous system, and increased anxiety and depression levels (Yanes and Reckelhoff, 2011; Maric-Bilkan and Galis, 2016).

The epidemiological studies mentioned above support our analysis. It is noteworthy that differences reported by ADEs in the two databases could be attributed to lifestyle and genetic (ethnic) differences between Japan and Western countries. Hypertension, a multifactorial disease influenced by environmental and genetic factors, shows differing prevalence and control rates across regions (Takeuchi et al., 2018). Japan has seen relatively lower improvements in hypertension awareness, treatment, and control compared to the United States and Europe (Sekikawa and Hayakawa, 2004). Despite higher obesity rates in European and American populations, East Asians are genetically more sensitive to salt and consume higher amounts of it (Bailly et al., 2020; Alfaras et al., 2016). Additionally, Japanese men’s lifestyle choices, such as excessive alcohol consumption and smoking, exacerbate hypertension (Kokubo, 2014). Interestingly, the indications for irbesartan were highly consistent across the two databases. Thorough analysis of these baseline data differences is crucial, as these demographic characteristics may introduce bias into ADE outcomes. Understanding these variations can enhance the interpretation of cohort differences and improve the accuracy of our findings.

### 4.2 SOC for which both databases satisfy the thresholds

#### 4.2.1 Metabolism and nutrition disorders

##### 4.2.1.1 Hyperkalemia

Pharmacological hyperkalemia is a predominant cause of elevated potassium levels in clinical practice (Kostis et al., 1996; Mahoney et al., 2005; Park et al., 2012; Antrobus et al., 1993). Although it may be asymptomatic, it can also pose life-threatening risks. Statistics reveal that approximately 1% of emergency department patients and 2% of hospitalized patients presenting with hyperkalemia succumb to the condition (Steiner et al., 2002). Multiple drugs induce hyperkalemia through various mechanisms, such as promoting transcellular potassium transfer or impairing renal potassium excretion. The inhibition of the RAS system, which reduces renal potassium excretion, is the primary mechanism by which drugs cause hyperkalemia (Ben Salem et al., 2014). Several clinical trials have shown that ARB therapy is associated with hyperkalemia, ranging from mildly asymptomatic to clinically significant and life-threatening levels (Mahoney et al., 2005; Palmer, 2003; Ramadan et al., 2005; Piner and Spangler, 2023).

A prospective, randomized controlled trial involving 244 elderly patients with mild-to-moderate essential hypertension demonstrated that low-dose irbesartan (150 mg/day) combined with spironolactone achieved better therapeutic efficacy with a lower risk of hyperkalemia compared to high-dose irbesartan (300 mg/day) (Chen Y. et al., 2018). A randomized controlled clinical trial demonstrated that the incidence of hyperkalemia in the irbesartan treatment group was 1.9% (Lewis et al., 2001). Another retrospective cohort study indicated that patients treated with ARBs alone had a poorer renal prognosis [HR (hazard ratio) 1.31] and a higher risk of hyperkalemia (HR 1.17) compared to those treated with Angiotensin converting enzyme inhibitors (ACEIs) alone. Irbesartan, in particular, showed inferior renoprotective effects (HR 1.35) (Hsu et al., 2017). In addition to irbesartan, other ARBs have been clinically reported to cause hyperkalemia (Desai et al., 2007; Konstam et al., 2009). ARBs may impair renal potassium excretion by blocking angiotensin II binding to adrenoceptors, thereby interfering with adrenal aldosterone secretion (Shier et al., 1989; Raebel, 2012). Age, medications, and reduced renal function are additional factors that increase hyperkalemia risk in most patients. Clinical trials have reported hyperkalemia in up to 6% of patients treated with ARBs, and this percentage rises to 30% in high-risk patients (e.g., those with renal insufficiency or diabetes mellitus) (Park et al., 2012; Palmer, 2004; de Denus et al., 2006).

Hyperkalemia exhibited significant case numbers and strong signal values in both FAERS (n = 234, EBGM05 = 20.38) and JADER (n = 52, EBGM05 = 27.60). This ADE is listed in both Japanese and FDA drug labels. Given the potential for life-threatening consequences due to fatal arrhythmias, timely diagnosis and management of hyperkalemia are crucial (Ideguchi et al., 2016). Prevention is preferable to treatment; thus, medical practitioners should inquire about concomitant medications, diets, supplements, and salt substitutes that may cause hyperkalemia before prescribing irbesartan (Roscioni et al., 2012). Important considerations when initiating irbesartan therapy include obtaining estimates of glomerular filtration rate and baseline serum potassium concentration. Timely monitoring of serum potassium after therapy initiation can help prevent hyperkalemia. If hyperkalemia occurs, prompt recognition and effective treatment are essential to counteract the effects of potassium on the heart, redistribute potassium into cells, and eliminate excess potassium from the body (Raebel, 2012).

##### 4.2.1.2 Hyponatremia

Hyponatremia is the most common electrolyte disorder in clinical practice, occurring in 15%–30% of hospitalized patients. Although often mild and asymptomatic, it remains clinically significant due to its potential to cause substantial morbidity and mortality from osmotic cerebral edema and osmotic demyelination if improperly treated (Verbalis et al., 2013). Certain medications (e.g., diuretics, antidepressants, and antiepileptics) are known causes of asymptomatic or symptomatic hyponatremia. However, hyponatremia can also occur with medications used in daily practice, including newer antihypertensive drugs (Liamis et al., 2008). For instance, a middle-aged woman with heart failure and reduced ejection fraction developed severe hyponatremia while on valsartan, with serum sodium levels dropping to 117 mmol/L, which normalized to 140 mmol/L 2 weeks after discontinuation (Mohammed et al., 2022). Similar cases have been reported with telmisartan and in elderly Japanese patients treated with an ARB and a thiazide (Takayama et al., 2019; Yamada et al., 2014).

A previous study showed that angiotensin II type 2 receptor blockers increase the risk of hyponatremia by 4.097-fold (Correia et al., 2014). In animal experiments, AT1 receptor blockers reduced glomerular filtration rate by more than 50% and increased urinary sodium excretion tenfold in neonatal rats (Chevalier, 2012). The ARBs’ inhibition of AT1 receptors, which reduces tubular sodium reabsorption and aldosterone secretion, may explain the induction of hyponatremia (Yamada et al., 2014; Fuzaylova et al., 2020). More recently, a pharmacovigilance analysis of the Spanish Pharmacovigilance database revealed that hyponatremia [ROR: 18.6 (9.6–35.9)] occurred with disproportionality in association with irbesartan use (Estévez Asensio et al., 2024). Our study also identified hyponatremia as a positive signal in FAERS (n = 343, EBGM05 = 18.45) and JADER (n = 11, EBGM05 = 4.47), although it is not listed in the drug labels. While no cases of irbesartan-associated hyponatremia have been reported, vigilance is necessary for the potential risk of hyponatremia with irbesartan use.

##### 4.2.1.3 Other metabolic disorders

In both FAERS and JADER, hyperuricemia and acidosis have emerged as new and significant signals. Hyperuricemia is an evolving metabolic disorder associated with conditions such as hypertension, myocardial infarction, metabolic syndrome, and heart failure (Borghi et al., 2020). The relationship between ARBs and blood uric acid levels is currently debated. For instance, losartan has been shown to have a uric acid-lowering effect by acting on the uric acid transporter protein 1 (URAT1) in the renal proximal tubule, inhibiting URAT1-mediated tubular reabsorption of uric acid, thereby increasing uric acid excretion (Enomoto et al., 2002; Iwanaga et al., 2007). Conversely, azilsartan and olmesartan are associated with elevated blood uric acid levels (Iwanaga et al., 2007; Shiga et al., 2017), suggesting that the uric acid-lowering effect of ARBs is drug-specific rather than class-specific (Smink et al., 2012; Chida et al., 2015). Studies on irbesartan’s impact on serum uric acid levels have produced mixed results, with some reporting beneficial effects and others finding no significant impact (Chida et al., 2015; Dang et al., 2006; Würzner et al., 2001). This inconsistency may stem from differences in study design and baseline characteristics of the populations studied. Given that serum uric acid levels can influence cardiometabolic risk factors, it is crucial to elucidate the relationship between irbesartan and uric acid.

Additionally, reports have linked the use of sartans to acidosis by suppressing the acidification of distal renal units, leading to metabolic acidosis (Sakallı et al., 2014; Wesson et al., 2012). Recognizing the potential risk of acid-base disturbances during irbesartan administration is essential for better patient management.

#### 4.2.2 Cardiac disorders

Our findings indicate that “cardiac disorders” was a significant SOC in both databases. Among these, bradycardia and various arrhythmias (e.g., cardiogenic shock, first-degree atrioventricular block) had higher case report numbers. Previous studies have demonstrated that endogenous angiotensin II exerts a tonic inhibitory effect on cardiac vagal neurotransmission through presynaptic AT1 receptor stimulation. AT1 receptor blockers inhibit this mechanism, promoting acetylcholine release from vagal endings, which may explain the reports of bradycardia with irbesartan (Yamaki et al., 2013; Potter, 1982; Kawada et al., 2007; Chiu et al., 1989; Wong et al., 1991). Although arrhythmias are infrequently reported with irbesartan dosing, it is noteworthy that many arrhythmias (e.g., atrial arrest) are secondary to hyperkalemia, a common adverse drug reaction following irbesartan use (Ideguchi et al., 2016; Kovesdy, 2014). Additionally, some studies involving populations undergoing renal dialysis indicate that irbesartan may have limited efficacy in preventing specific cardiovascular events, such as atrial fibrillation and heart failure, with reports of symptomatic hypotension and renal dysfunction occurring more frequently (Yusuf et al., 2011; Disertori et al., 2009; Peters et al., 2014). Thus, it is essential to remain vigilant for potential cardiac issues following irbesartan administration and to manage them promptly.

#### 4.2.3 Renal and urinary disorders

Although irbesartan has potential renoprotective effects, such as reducing inflammation and lowering urinary protein (Lewis et al., 2001; Zhong et al., 2020; Parving et al., 2001; Deferrari et al., 2002), ARB use may also increase the risk of acute kidney injury (Wynckel et al., 1998; Toto et al., 1991). Our study identified signals such as acute kidney injury (listed in the drug label) and tubulointerstitial nephritis (new signal). While angiotensin II is believed to cause local ischemia and inflammation in the kidneys, it may also have renoprotective and beneficial effects by enhancing the myogenic response to changes in stress (Benndorf et al., 2009; Griffin and Bidani, 2009). Long-term ARB treatment can lead to an increase in plasma aldosterone concentrations to pre-treatment levels, resulting in “aldosterone escape,” which can cause glomerular and tubular fibrosis (Hubers and Brown, 2016). Angiotensin II receptor antagonist-induced acute renal failure may occur in patients sensitive to reduced renal blood flow (Lee and Kim, 2001). A significantly increased risk of end-stage renal disease following losartan treatment and acute interstitial nephritis after valsartan treatment has been reported, potentially counteracting the renoprotective effects of ARBs (Chen et al., 2019; Miao et al., 2011).

Irbesartan treatment can also cause potential renal injury. In a trial of heart failure treatment with preserved ejection fraction, patients receiving irbesartan experienced a greater decrease in estimated glomerular filtration rate and a higher likelihood of worsening renal function compared to those receiving a placebo (8% versus 4%) (Metra and Lombardi, 2014). Similar renal injuries have been reported in studies by Damman et al. (2016), Veelken et al. (1998), and Metra and Lombardi (2014) The results of a prospective, double-blind, multicenter trial investigating the treatment of severe hypertension indicated that the incidence of elevated blood creatinine levels was 3.0% in patients treated with irbesartan/hydrochlorothiazide and 1.8% in those receiving irbesartan alone (Neutel et al., 2009). These findings align with our observation of a potential association between irbesartan and renal injury. Therefore, we advocate that all patients treated with irbesartan undergo a complete renal and renal vascular ultrasound (to rule out vascular stenosis), and that renal function be dynamically monitored during the course of medication, especially in patients with renal hypoperfusion (Descombes and Fellay, 2000).

### 4.3 SOCs that meet thresholds in FAERS only

#### 4.3.1 Nervous system disorders

Syncope has the highest number of reported cases (n = 151, EBGM05 = 4.30) under “nervous system disorders” in FAERS and is a new signal. Previous studies have shown that vagal excitatory events, including syncope, are associated with AT1 receptor blockers during the treatment of essential hypertension (Sever and Hughes, 2001; Lovelace et al., 2023). This blockade eliminates the modulatory effect of angiotensin II on the Bezold-Jarisch reflex (Lovelace et al., 2023). Additionally, a large double-blind randomized clinical trial demonstrated an increased risk of syncope with the combination of telmisartan and ramipril compared to ramipril alone (Yusuf et al., 2008). It is noteworthy that baroreflex sensitivity decreases with age and systemic hypertension, making older hypertensive patients more susceptible to syncope, which aligns with the age profile in FAERS (nearly half of the reports were from patients ≥65 years of age) (Albasri et al., 2021). In a prospective, observational, descriptive, multicenter clinical study, the highest rate of neurological adverse events was reported following irbesartan treatment, at 1.62% (50 adverse drug events reported), which supports our findings (Ihm et al., 2019).

#### 4.3.2 Vascular disorders

All antihypertensive medications may predispose older patients to symptomatic orthostatic hypotension (Aronow, 2009). Orthostatic hypotension is characterized by an abnormally large drop in blood pressure upon standing, increasing the risk of adverse outcomes (Wieling et al., 2022). With age, left ventricular compliance decreases, ventricular wall thickness increases, left ventricular diastolic filling decreases, and diastolic function is impaired. Therefore, older hypertensive patients are more likely to develop orthostatic hypotension, consistent with our baseline information analysis (Albasri et al., 2021). Other potential mechanisms include inhibition of bradykinin catabolism by ARBs, anaphylaxis induced by acute mast cell degranulation, or overtreatment (Nielsen, 2005). A clinical study involving 9016 patients with a mean follow-up of 4.1 years found that more patients in the irbesartan group than in the placebo group developed symptomatic hypotension (127 versus 64 patients) (Yusuf et al., 2011). Additionally, a meta-analysis showed that perioperative continuation of ARBs was associated with an approximately 30% increase in intraoperative hypotension incidence (Hollmann et al., 2018). Considering that orthostatic hypotension greatly reduces quality of life and may cause disability, syncope, and traumatic injuries, patients on irbesartan should be adequately informed of the potential risk of hypotension and be encouraged to adopt simple lifestyle measures (moderate, non-strenuous activity, slow changes in posture, etc.) (Ricci et al., 2015).

#### 4.3.3 Hepatobiliary disorders

Pharmacological liver injury is a potential complication of almost all prescribed medications, including irbesartan (Guo et al., 2005). Initially, manufacturers of irbesartan did not recognize any association between the drug and severe liver dysfunction (Hariraj et al., 2000). However, case reports have emerged indicating otherwise. One such case involved a 56-year-old man treated with irbesartan 300 mg/day for 8 days, who was subsequently admitted with jaundice. After excluding other etiologies, cholestatic hepatitis due to irbesartan was diagnosed (Andrade et al., 2002). Another case involved a 62-year-old woman admitted with jaundice after 1 week, having been treated with irbesartan 300 mg/day for a month. Examination revealed jaundice, hepatomegaly, and a liver biopsy showing bile duct dilatation and cholestasis (Hariraj et al., 2000). These findings align with our study.

Current understanding suggests a combination of drug-induced and immune-mediated hepatic injury associated with irbesartan (Annicchiarico and Siciliano, 2005). The hepatotoxicity mechanism likely involves metabolic mediation, with genetic variants in AT1RA metabolism producing reactive metabolites via hepatic cytochrome P450, predisposing patients to drug hepatotoxicity (Andrade et al., 2002). Similar hepatotoxicity has been reported with other sartan drugs (Zahedi et al., 2023; Bosch, 1997a; Vallejo et al., 2000; González-Jiménez et al., 2000; Odak et al., 2021; Basile et al., 2003). In the United States, drug therapy has become a leading cause of acute liver failure (Lee, 2003). Therefore, patients with pre-existing liver disease should be informed of these risks, and immediate discontinuation of the drug with clinical follow-up may be necessary to prevent serious liver damage.

### 4.4 SOCs that meet thresholds in JADER only

#### 4.4.1 Musculoskeletal and connective tissue disorders

In the JADER database, the SOC “musculoskeletal and connective tissue disorders” met all the algorithmic signal values positively. The number of reported cases of rhabdomyolysis (n = 22, EBGM05 = 5.39) was second only to hyperkalemia. Interestingly, rhabdomyolysis is listed in the Japanese drug label but not in the FDA drug label. Rhabdomyolysis involves catabolic necrosis of muscle tissue and the release of intracellular contents into the bloodstream, caused by various mechanisms, including drugs and toxins, and can be life-threatening in severe cases (Cabral et al., 2020). A common feature of rhabdomyolysis is a decrease in myoplasmic ATP paralleled by a sustained increase in cytoplasmic Ca2+ concentration (Hohenegger, 2012). Irbesartan is primarily metabolized by the liver, and some of its metabolites may exert toxic effects on skeletal muscle cells. By interfering with oxidative phosphorylation in myocytes, the drug or its metabolites can cause mitochondrial dysfunction, increase the production of reactive oxygen species, damage cell membranes and organelles, and ultimately lead to myocyte necrosis and lysis (Bonora et al., 2019). Furthermore, irbesartan controls blood pressure mainly through the RAAS; an imbalance in RAAS regulation may result in inadequate muscle perfusion, particularly in patients with renal insufficiency or other underlying conditions, predisposing myocytes to ischemic necrosis (Sunaga and Ryo, 2022). Irbesartan may also elevate the incidence of rhabdomyolysis by interfering with the metabolic pathways of statins, leading to statin accumulation in the body (Kiaie et al., 2021). Lastly, irbesartan may indirectly increase the risk of rhabdomyolysis by inducing electrolyte disturbances, such as imbalances in potassium ion concentration.

A case-control study based on a Japanese population identified ARB as a risk factor for rhabdomyolysis associated with statin administration (Hashiguchi et al., 2018). Although there is no direct evidence of irbesartan-induced rhabdomyolysis, it is important to note that rhabdomyolysis can exacerbate renal injury through hypovolemia, myoglobinuria, and metabolic acidosis, potentially amplifying the renal injury effects of irbesartan (Chavez et al., 2016). In the event of rhabdomyolysis, it is crucial to discontinue the medication, perform prompt fluid replacement, correct electrolyte abnormalities, and, if necessary, initiate continuous renal replacement therapy (Chavez et al., 2016).

### 4.5 Other signals

#### 4.5.1 Acute pancreatitis

Acute pancreatitis is a leading cause of gastrointestinal-related hospitalizations, with a mortality rate of approximately 30% in critically ill patients (Tu et al., 2012). RAS inhibitors have been associated with an increased risk of acute pancreatitis (Eland et al., 2006; Birck et al., 1998; Bosch, 1997b). The pancreas contains a localized RAS with angiotensin II receptor subtypes AT1 and AT2 found in pancreatic ducts, blood vessels, and adenohypophysial cells, involved in the physiological regulation of digestive enzyme secretion (Famularo et al., 2005). It is hypothesized that bradykinin may contribute to the pathogenesis of acute pancreatitis through its role in the RAS and the bradykinin-kinin system (Bas et al., 2007; Hirata et al., 2002). Two cases of acute pancreatitis have been reported in patients exposed to irbesartan (Famularo et al., 2005; Fisher and Bassett, 2002). Irbesartan may contribute to the development of pancreatitis by triggering pancreatic cell dysfunction through alterations in local hemodynamics or by directly impacting metabolic pathways within pancreatic cells (Mayerle et al., 2019). Furthermore, angiotensin II receptor blockers, including irbesartan, have the potential to induce metabolic disturbances that affect lipid metabolism, insulin sensitivity, and glucose metabolism. Given that metabolic syndrome is a recognized risk factor for pancreatitis, irbesartan may indirectly elevate the risk of pancreatitis by influencing insulin metabolic pathways (Badalov et al., 2007). Additionally, there is a hypothesis suggesting that irbesartan may induce cholestasis by affecting the biliary system (this signal is also indicated in Figure 4A) (Maleszka et al., 2017). This condition may further contribute to biliary pancreatitis, particularly in patients with a history of gallstones or biliary tract disease. Given the limited number of studies examining the association between acute pancreatitis and irbesartan, the new signals of acute pancreatitis that we observed should be interpreted with caution and warrant further investigation in future studies. Nonetheless, clinicians should consider pharmacological pancreatitis in irbesartan-treated patients presenting with severe, unexplained abdominal pain (Famularo et al., 2005).

#### 4.5.2 Cutaneous adverse reaction

Our analyses identified several irbesartan-associated ADEs affecting the skin, including “rash maculopapular” (n = 25, EBGM05 = 2.80), “toxic skin eruption” (n = 25, EBGM05 = 6.00), and “pemphigoid” (n = 23, EBGM05 = 8.14). Although cutaneous adverse reactions to ARBs are uncommon (Steckelings et al., 2001; Palleria et al., 2019), the number of cases in our analysis is significant. Of the seven case reports of irbesartan-induced rash, there was one case each of erythema multiforme (Constable et al., 2006), lichenoid rash (Pfab et al., 2006), and pruritic erythematous papules (Cardoso et al., 2019), and four cases of purpuric rash (Foti et al., 2014). Gambini et al. reported a case of irbesartan-induced maculopapular rash in a patient who developed an acute febrile reaction 5 days after starting irbesartan (Gambini et al., 2003). Vena et al. described eczema-like reactions to irbesartan, suggesting that these reactions were related to interference with the kallikrein-kinin system and elevation of circulating and cutaneous pro-inflammatory bradykinin peptides, as ARBs can increase bradykinin levels in hypertensive patients (Campbell et al., 2005; Vena et al., 2013). Additionally, a positive lymphocyte transformation test in patients with rashes suggests an immune mechanism, classifying it as an allergic reaction (Cardoso et al., 2019). In this case, irbesartan may induce a hypersensitivity reaction by stimulating the production of specific antibodies in the body. Upon re-exposure to irbesartan, the drug can bind to these antibodies, activating mast cells and basophils, which results in the release of histamine and other inflammatory mediators. The release of histamine can cause vasodilation and increased vascular permeability, potentially leading to the development of a rash. Additionally, there is a possibility that irbesartan may directly induce mast cell degranulation, resulting in the release of histamine independent of the immune pathway (Nielsen, 2005). This mechanism is typically associated with individual susceptibility and may lead to nonspecific papular urticaria. Recent investigations into cutaneous adverse reactions associated with irbesartan have employed spontaneous reporting databases. A study by Sridharan K et al. identified a potentially increased risk of angioedema with losartan (ROR 3.6 [3.3, 3.8]) and irbesartan (ROR 2.4 [2.1, 2.7]) in comparison to other ARBs, as demonstrated in a pharmacovigilance analysis utilizing the FAERS. Furthermore, Viola E et al. reported a significant disproportionate signal for irbesartan-associated photosensitivity (IC025: 0.62) based on data from VigiBase (Sridharan and Sivaramakrishnan, 2024). Our analysis of FAERS data corroborates these findings, revealing that angioedema and skin photosensitivity were reported in asymmetric proportions among the ADEs related to irbesartan, thereby reinforcing the conclusions of the aforementioned pharmacovigilance studies (Viola et al., 2015).

#### 4.5.3 Agranulocytosis

Our study suggests that several hematological adverse reactions not listed in the specifications may be associated with irbesartan use, including “agranulocytosis” (n = 34, EBGM05 = 4.93) and “bicytopenia” (n = 5, EBGM05 = 4.65). Hematological toxicity has been reported in 0.3% of the treated population due to irbesartan use. This rare but potentially serious adverse reaction is particularly concerning in older adults receiving multiple medications (Gómez-Sayago et al., 2012). An 85-year-old woman developed leukopenia after treatment with irbesartan 150 mg/day, with the Karch-Lasagna algorithm used to demonstrate this causality (Gómez-Sayago et al., 2012). Another retrospective investigation of 184 dialysis patients found that 4–6 weeks after starting treatment with losartan (50 mg/day), the mean hemoglobin concentration decreased from 118 g/L to 101 g/L (Schwarzbeck et al., 1998). Similarly, there were “susceptible” patients whose hemoglobin levels decreased significantly after irbesartan treatment (Simonetti et al., 2007). Irbesartan may contribute to anemia through direct inhibition of erythropoietin or insulin-like growth factor-1 production, or indirectly by improving renal perfusion and subsequently reducing oxygen consumption (Afzali et al., 2006). A negative effect on hematopoiesis at the bone marrow level has also been proposed due to the discovery of AT1 on progenitor cells (Ersoy et al., 2005). These findings may partially explain the new signals we identified. Therefore, close monitoring of hemoglobin and white blood cell counts is recommended when treating patients with severe renal insufficiency with sartans (Schwarzbeck et al., 1998).

In summary, the identification of these new signals necessitates a reassessment of irbesartan prescribing practices, particularly for specific patient populations such as those with pre-existing renal impairment and the elderly. Careful consideration is required before initiating therapy, including regular assessments of renal function and vigilant monitoring for signs of acute pancreatitis or agranulocytosis. Furthermore, the study’s findings support a recommendation to update the drug labeling for irbesartan, informing prescribers of these newly identified risks and emphasizing the importance of monitoring at-risk populations to enhance patient safety. Lastly, our results contribute to the development of updated clinical guidelines for irbesartan, incorporating recommendations on risk stratification, monitoring strategies, and patient counseling to ensure awareness of potential risks among healthcare professionals and patients alike.

### 4.6 Time to onset

In drug safety evaluation, it is crucial to assess the interval between drug administration and the onset of ADEs. This assessment can elucidate the underlying mechanisms of ADEs, identify specific time windows of risk during treatment, and facilitate earlier prevention or diagnosis of adverse reactions (Leroy et al., 2014). The timing of ADEs following RAS inhibitor use has been previously reported. For instance, angioedema after ACEI use typically occurs within the first week of treatment, although a significant proportion can manifest after months or even years (Howes and Tran, 2002). Diseases such as pemphigus associated with ARB therapy often appear months or years after treatment initiation (Steckelings et al., 2001).

A study by Zahedi I et al. suggests that liver function enzymes should be monitored in high-risk patients from the initiation of losartan, from a few days to several months (Zahedi et al., 2023). In our study, the median TTO of “hepatobiliary disorders” and “skin and subcutaneous tissue disorders” was 195 and 46 days, respectively, aligning with the clinical studies mentioned. Additionally, we found that the adverse effects of irbesartan predominantly occurred at 1 month (34.14%) and 1 year (32.32%) after administration. Acute hypersensitivity or immune-mediated reactions, such as angioedema or anaphylactic rash, may occur early in irbesartan therapy, supported by median onset times of 47 days for “skin and subcutaneous tissue disorders” and 17 days for “immune system disorders” in our TTO analysis. Early drug interactions, especially with concurrent medications, may lead to increased irbesartan concentrations, causing adverse reactions like hypotension and dizziness (Nunnery and Mayer, 2019). Long-term use can gradually impair renal function, particularly in patients with chronic kidney disease, as indicated by a median onset of 161 days for “renal and urinary disorders”. Furthermore, long-term therapy may result in drug tolerance or adaptation, leading to an increased frequency of adverse reactions over time (Brass, 1984). These findings highlight the need for clinicians to closely monitor patients in the early stages of treatment to promptly detect immune reactions or organ damage. Additionally, regular assessments of renal and hepatic function, as well as electrolyte levels, are essential during long-term therapy, especially beyond 1 year, to prevent the progression of chronic adverse reactions. In summary, our findings underscore the necessity for continuous vigilance in clinical practice (Maignen et al., 2010).

### 4.7 Limitations

It is important to acknowledge the limitations of our study:

1. Differences in case reports: The total number of cases in the two databases varied significantly; JADER was limited to case reports, whereas FAERS included periodic reports that encompassed non-serious cases over an extended period. This discrepancy may have influenced the results of our analyses (Zou et al., 2024).
2. Missing key variables: Despite conducting subgroup analyses, some critical variables, such as age and sex, were missing from the data. This absence could have impacted the results, alongside issues related to underreporting and overreporting, which may introduce bias into the findings (Zhang Y. et al., 2024).
3. Lack of comorbidity data: We were unable to obtain information on patients’ comorbidities, preventing us from excluding reports that may represent high-risk factors for certain ADEs (Zhang X. et al., 2024).
4. Signal assessment limitations: Our differential analyses were restricted to assessing the strength of signals related to ADEs, which did not permit quantification of risk or identification of drug-related causality. Consequently, prospective studies are necessary to validate the signals associated with newly identified ADEs (Wu et al., 2024).
5. Confounding variables: Although we employed sensitivity analyses, the presence of confounding variables—such as dosage administered, duration of use, comorbidities, and polypharmacy—may affect the accuracy of our results (Wang et al., 2024b; Wei et al., 2024).
6. Generalizability of findings: This study primarily focused on data from two databases representing the United States and Japan, which may limit the generalizability of the findings to other populations with differing demographic characteristics, health practices, and prescribing patterns.

Despite these limitations, the combined FAERS and JADER databases provide valuable resources for post-marketing safety monitoring of irbesartan.

## 5 Conclusion

This study represents the first comprehensive and systematic analysis of ADEs associated with irbesartan, utilizing data from the FAERS and JADER databases. Signal detection at the SOC level identified three SOCs with significant signal strength in both databases: “metabolism and nutrition disorders,” “cardiac disorders,” and “renal and urinary disorders.” Common adverse events such as “acute kidney injury,” “hyperkalemia,” “bradycardia,” and “hypotension” were consistent with the drug label. Additionally, we identified new signals, including “acute pancreatitis,” “rhabdomyolysis,” “maculopapular rash,” “pemphigoid,” and “agranulocytosis.” Moreover, we provide a detailed timeline for the onset of ADEs. This pharmacovigilance assessment not only enhances our understanding of irbesartan’s safety profile but also offers valuable insights for future research and informs clinical practice.

## Data Availability

The original contributions presented in the study are included in the article/Supplementary Material, further inquiries can be directed to the corresponding author.

## Supplementary material

SUPPLEMENTARY FIGURE S1 Volcano plots were generated to display positive risk signals for irbesartan in FAERS **(A)** and JADER **(B)** databases, with the horizontal axis representing log2-transformed ROR values and the vertical axis displaying −log10 of Bonferroni-adjusted *P*-values. Statistically significant signals are highlighted in color. In the FAERS and JADER databases, the top 20 and top 10 signals were labelled ranking by log2 ROR values, respectively. The *P*-values were adjusted using the Bonferroni method.

SUPPLEMENTARY FIGURE S2 Subgroup analysis was performed to identify the top 15 signals at the preferred term level, stratified by sex and age groups in the FAERS and JADER databases. Sex-based subgroup analyses were conducted for FAERS **(A)** and JADER **(E)**, with Venn diagrams **(B, F)** illustrating the intersection of signals between male and female subgroups. Age-based subgroup analyses were conducted for FAERS **(C)** and JADER **(G)**, with Venn diagrams **(D, H)** showing the intersection of signals between the <65 years and ≥65 years age groups. Arrows indicate instances where the lower bound of the 95% confidence interval for the ROR exceeds 30. Due to the limited number of positive signals in the JADER database, E and G displayed fewer than 15 positive signals.

SUPPLEMENTARY TABLE S1 The 219 PT entries simultaneously satisfying the four methods of disproportionality analysis with positive signal values in FAERS, displayed in descending order of case number. Asterisks (*) indicate new signals not listed in the drug label. The *P*-value is adjusted with Bonferroni correction method.

SUPPLEMENTARY TABLE S2 The 20 PT entries simultaneously satisfying the four methods of disproportionality analysis with positive signal values in JADER, displayed in descending order of case number. Asterisks (*) indicate new signals not listed in the drug label. The *P*-value is adjusted with Bonferroni correction method.

SUPPLEMENTARY TABLE S3 The results of the sensitivity analysis identified positive ADEs for irbesartan using the FAERS data, specifically excluding cases involving common co-administered drugs at the PT level. This approach ensured that the signals detected were directly attributable to irbesartan, thereby improving the reliability of the findings related to its safety profile. ADEs: adverse drug events; PT, preferred term.

SUPPLEMENTARY TABLE S4 Detailed TTO analysis results at the SOC level. min: minimum; max: maximum; IQR: interquartile range; q1: quartile 1/4; q3: quartile 3/4; SD: standard deviation; SE: standard error.
