# Supplementary figures and images for: A real-world pharmacovigilance analysis of adverse events associated with irbesartan using the FAERS and JADER databases
Source: Front Pharmacol. 2024 Nov 20;15:1485190. doi: 10.3389/fphar.2024.1485190 (PMC11614654; doi:10.3389/fphar.2024.1485190)

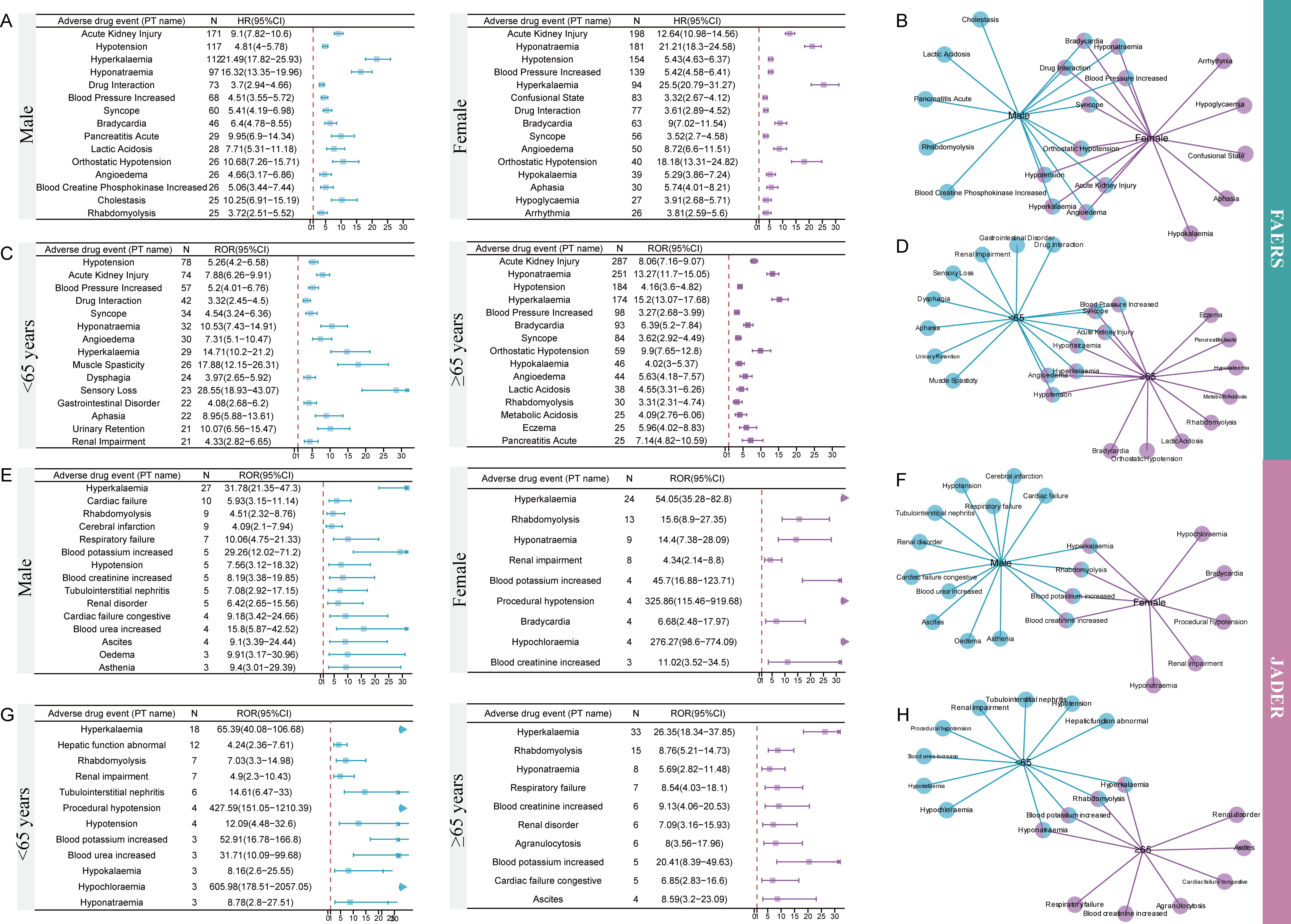

Supplement: Supplementary file 3 [file Image2.TIF]

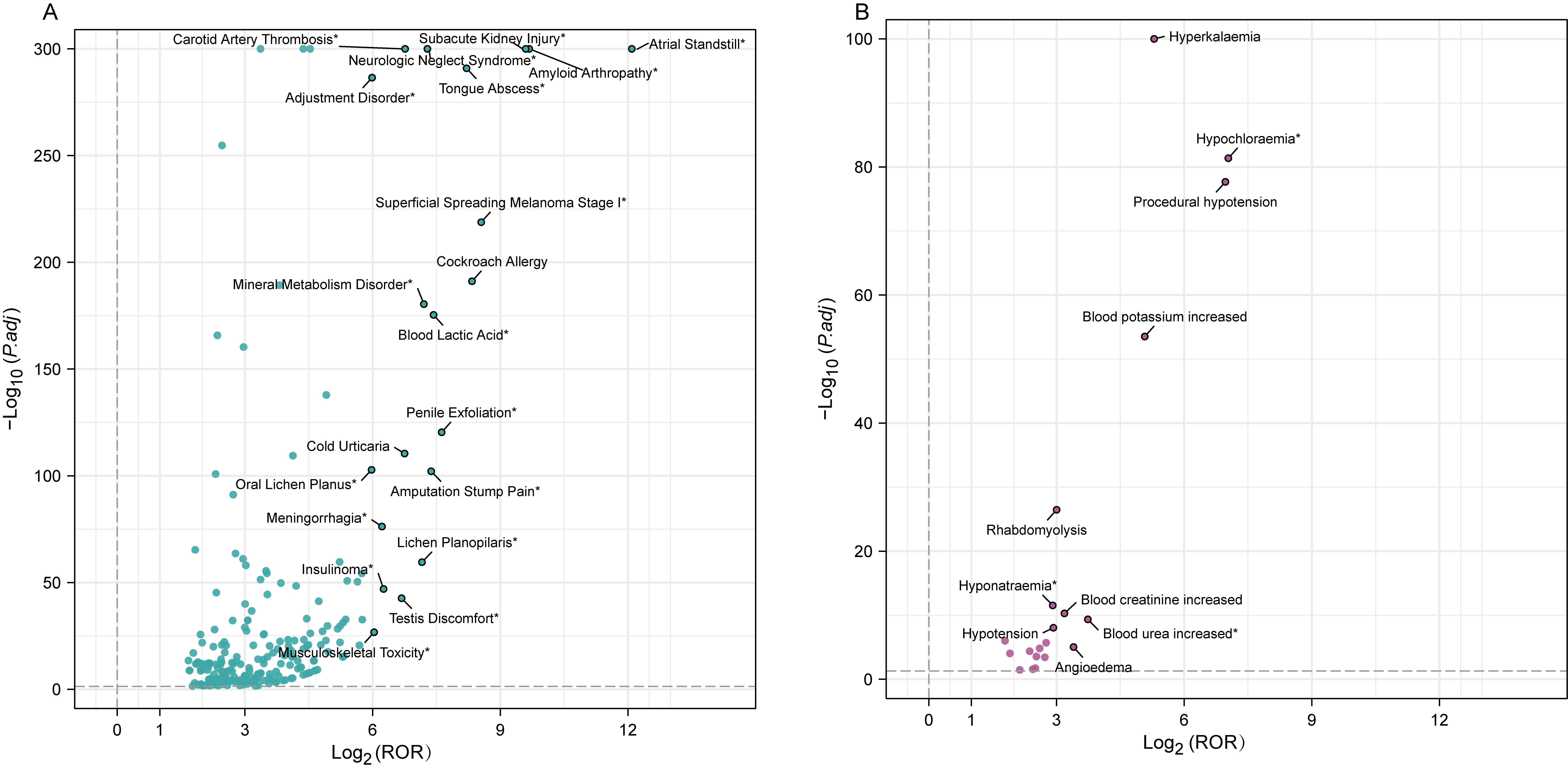

Supplement: Supplementary file 4 [file Image1.TIF]
